# Inaugural editorial post COVID-19 global society: issues, challenges and edging forward

**DOI:** 10.1007/s44282-023-00001-z

**Published:** 2023-03-13

**Authors:** Rajendra Baikady

**Affiliations:** 1 Central University of Kerala, Kasaragod, India; 2 University of Johannesburg, Johannesburg, South Africa; 3 York University, Toronto, Canada

## Abstract

The COVID-19 pandemic outbreak in the later part of 2020 resulted in enormous social and economic disruption leading to a quick transformation of global society. A health crisis that interrupted the lives of every individual across the globe, soon changed in to a human, economic and social crisis. All segments of the society irrespective of their status and privilege experienced the challenges posed by COVID-19 pandemic, however the marginalized and vulnerable populations including people living in poverty situations, older persons, persons with disabilities, youth, and indigenous peoples in developing, less developed and lower- and middle-income countries had the greater impact on their lives and livelihood. Furthermore, homeless population, people effected by war and conflict, people without access to running water, refugees, migrants, or displaced population continues to face the aftermaths of the pandemic.

## How COVID-19 impacted global society

The pandemic resulted in tremendous human suffering with serious and long-term implications for people’s health, well-being and quality of life. It did not only bring in public health issues to the global population but also brought in issues related to social interconnectedness, subjective well-being and stress, mental health, personal security, financial vulnerability, lack of trust in social institutions, relationships and administrations and modern medical systems. Inequality and social disparity were at high in developing and less developed countries even before the outbreak of the pandemic. However, the factors such as job loss, salary cut, lack of employment opportunities, lockdown and social restrictions during the pandemic further deteriorated the economic condition of overall population. The gap between the rich and poor elaborated, while poor experiencing several adverse effects of the pandemic that might not be experienced by economically advanced population. In terms of death and loss of lives, even well-developed countries with advanced medical systems were not able to save lives. Death rates increased in an alarming way and the globe watched it in horror and shock. These death rates in the developed countries in the west revealed the untold stories of health disparity and economic vulnerability. The pandemic exposed a large number of populations across the nation states to unexpected trauma, shock and fear of loss, anxiety and grief. The unknown fear of loss and anxiety resulted in social isolation, increased visit to hospitals, increase in violence, crime and other social problems. While one section of the population who were privileged to avail hospital facilities in both developed and developing countries rushed to hospitals with hope to save theirs and their loved one’s lives. On the other hand, poor and underprivileged population who could not offered hospital care and medication depended on home remedies and community care. These trends further give an idea about the deprivation and inequality persisting in our global society.

## COVID-19 and our lives

Covid 19 pandemic has profoundly changed our lives. On the one hand Millions of people lost their lives, and another large section of population lost their livelihood and employment. Societies across the globe started witnessing widened inequalities, undermined progress on global poverty and lack of clean energy, food and health care. Social impact of the pandemic resulted in weakening social connections and relationships, trust issues in relationships among the peoples which resulted in the social and communal harmony. COVID-19 pandemic also revealed the amount of digital inequality or digital divides existed between the population. With the lockdowns/ restrictions imposed on public movement and social life, people relayed on internet for buying food and work from home, where as educational activities across the world moved online from traditional teaching and learning. However, the effectiveness of these activities depends on the availability of infrastructure, internet connectivity and the speed, computer competency or the skills required for using computer and internet devises effectively and efficiently. However available literature documents great difficulties experienced by rural populations in accessing online facilities during COVID-19 pandemic [1, 2]. Further student community experienced the most deprivation in terms of digital divide as studies pointed out there were communication and participation issues in online learning under COVID-19 [3]. In terms of Youth population, they witnessed the school closure, lack of infrastructure facilities to continue online or distance learning and most importantly missing social life among the peer groups. Further a large number of youth and children, especially girls were under the threat of discontinuing the schooling in the post covid era.

Health care facility and availability of health care, especially for the poor section of the society was another concern during the COVID-19 pandemic. On the one hand health care facility to prevent and promote health was available through various measures such as wearing a mask, using hand sanitizer, washing hand and maintain social distance in crowded areas. However, health inequality or the disparity in terms of health acre accessibility resulted in deteriorated health of poor and underprivileged section across the globe. In countries where health care is largely privatized had the issues of overcharging and irrational cost. Where as developed countries with advanced health care facilities had fewer resources such as hospital beads and human resource to support and provide health care. Hence saving lives was a major challenge across the globe. Available literature also reviles that the deprived and underprivileged communities witnessed more deaths than the any other section of the population [4–7] In terms of employment millions lost their working opportunity while worst hit are workers in the informal economy, young people and women. The pandemic has had a disproportionate impact on the people living with low income in low- and middle-income countries.

## Building back a better, sustainable and equal society

United nations in the year 2015 formulated and adopted 17 Sustainable Development Goals, also called as global goals to accelerate the development of global economy, reduce inequality, poverty, bring in gender equality, strengthen social institutions and overall wellbeing. However, outbreak of COVID-19 resulted as an obstacle for achieving the sustainability goals by 2030. COVID-19 pandemic disrupted several areas where considerable progress was achieved by several countries irrespective of their financial status and availability of resources. One such destruction is widening gender inequality. SDGs aimed for considerable reduction in gender inequality by 2030. However, the pandemic resulted in widening gender gap in terms of increased drop in employment opportunities for women, increased working hours, invisible and unpaid work and lack of protection and safety [8]. Hence the post COVID pandemic society need to intensify initiatives to empower women through social security, women friendly employment policies and inclusive social policy initiatives. Another major area where COVID-19 had its impact is poverty reduction. Impact of COVID-19 on Social, economic and political aspects in global society resulted in increased poverty and disparity in global society. Loss of employment, increased medical expenditure, loss of family income due to death of earning members in the family and closure of business and small-scale industries led to increased debt and financial disparity. Thus, making the goal of achieving poverty free society by 2030 a non-achievable dream for many countries in the globe. In order to tackle this issue development programmes, economic investment and the social security policy in the post pandemic era should make minimizing poverty arising from COVID-19 as an important policy priority. Further priority should also be given to engaging unemployed youth and populations in economic development, while ensuring safety, social security and decent working condition of existing workforce.

Building strong institutions, facilitating peace and inclusive growth, developing and maintain strong international collaboration and coordination are also the priorities of post COVID-19 development to build a strong global society. In a world with widening inequality and disproportion of resources it is reasonable to build international cooperation where poor countries in the global south can avail the support and guidance from the developed economies. In order to reduce the power imbalance and exploitation by the western economies the government and administration in the global south need to find ways and means in developing and implementing mutually beneficial international collaboration. Further development in the post COVID society should also focus on enhancing all the four domains of health, including physical, psychological, social, and spiritual domains, thus contributing to the Sustainability Goal of health for all by 2030.

## About discover global society

Societies across the globe are undergoing rapid growth and transformation. The processes of globalization, neoliberal market, and technological advancement has resulted in unprecedented social change and development. Changing social norms, values, and ethical issues in a contemporary global society demands further investigations of several social parameters to better understand the societal challenges while working towards a more sustainable future. *Discover Global Society* is an international, inclusive journal that provides opportunities to educators, researchers, and scientists across various social science disciplines for rapid dissemination of their research findings related to key societal issues and challenges. Showcasing authorship from diverse backgrounds and covering perspectives from both the global south and north, *Discover Global Society* includes articles that will influence social policy at different levels.

*Discover Global Society* is an international and interdisciplinary journal that aims to publish the finest research findings on the complexities of modern global society and its development and challenges. While open to a variety of perspectives, we particularly encourage scholarship that examines the effects of globalization, neoliberalism, and growing internationalism on different aspects of societies and their challenges in different regions and regimes. Further research that examines the intersection of social, political, and economic aspects of the growing societal concerns within intercultural communities and societies are particularly encouraged. The journal is open to different methodological and theoretical approaches and seeks to publish articles that appeal to a wide readership, including policymakers and practitioners.

## Topics

*Discover Global Society* covers a broad range of topics related to different societal issues, development challenges, and current transformations. Research on societies at the local, national, and international levels around the world are encouraged. Research findings contributing to the advancement of the UN Sustainable Development Goals (SDGs) are particularly encouraged. Specific topics covered by *Discover Global Society* includes (but not limited to).

- Social problems
- Social change and social issues
- Complexities of modern and globalized societies
- Movement of people
- Global infrastructure
- Sustainability
- Urbanization
- Global governance and issues related to governance
- Public policy
- Social policy and welfare
- Social security
- Globalization
- Culture
- Inequality
- Poverty
- Development and discrimination
- Gender
- Nationalism
- Religion
- Ethnicity
- Migration
- Terrorism and criminal activities
- Genocides
- Health and environmental degradation
- Youth and adolescent issues
- Disability
- Community development
- Social development
- Social policy and social work

## Edging forward

COVID-19 pandemics has given us an opportunity to accelerate the research, teaching and practice in the areas of social problems, social change and inequality. Learning from various interventions, experiments, loss and gains during the past three years need for comprehensive, systematic, evidence based and participatory social policy in a post COVID-19 society is advocated by both researchers, practitioners and academics across the disciplines and geographical boundaries. If not addressed through evidence-based policies, the inequality, exclusion, discrimination and global unemployment produced by the pandemic may impact global society both in the medium and long term. Furthermore, administrations in each country should make sure that vulnerable and marginalized sections of the society are well covered by comprehensive and universal social protection measures to fight against and overcome shocks posed by pandemic. Well-coordinated governance, protecting left behind people and places, and supporting small businesses and vulnerable workers should be the policy priorities in the post pandemic era. Both state and non-state initiatives in bringing back social and economic normality in post covid society can not be overlooked. Learning from the lessons of pandemic administration should work towards building more resilient and long-term support system to achieve overall wellbeing while focusing on the upliftment of marginalized and vulnerable population. Greater thrust is required in ensuring job security, improving poor quality housing, environmental quality, intervening with mental and other health challenges and social isolation. In order to achieve the sustainable development goals and overall wellbeing of global population, *Discover Global Society* seek out to promote high quality, evidence-based research findings on all aspects of global society.

## Data Availability

Not applicable.
